# Aberrant Expression of Beclin-1 and LC3 Correlates with Poor Prognosis of Human Hypopharyngeal Squamous Cell Carcinoma

**DOI:** 10.1371/journal.pone.0069038

**Published:** 2013-07-25

**Authors:** Juan Wang, Xin-Liang Pan, Li-Jie Ding, Da-Yu Liu, Da-Peng Lei, Tong Jin

**Affiliations:** 1 Department of Otolaryngology, Qilu Hospital of Shandong University, Jinan, Shandong, China; 2 Department of Epidemiology and Biostatistics, School of Public Health, Shandong University, Jinan, Shandong, China; Kyushu Institute of Technology, Japan

## Abstract

**Background:**

Beclin-1, a key regulator of autophagy. Microtubule-associated protein 1 light chain 3 (LC3), is involved in autophagsome formation during autophagy. The autophagic genes beclin-1 and LC3 paly an important role in the development and progression of tumor. This study was designed to investigate the expression of beclin-1 and LC3 and to clarify their clinical significance in hypopharyngeal squamous cell carcinoma (HSCC).

**Methods:**

Eighty-two surgical hypopharyngeal squamous cell carcinoma specimens and fifty-four adjacent non-cancerous mucosal epithelial tissues were obtained. Beclin-1 and LC3-II expression was examined by immunohistochemistry, real-time RT-PCR and Western blotting assays. Correlations with patient clinical characteristics and overall survival were evaluated.

**Results:**

Beclin-1 was positively expressed in 42.7% (35/82) of HSCC specimens (adjacent non-cancerous tissues, 79.6%, 43/54; *P*<0.0001). Furthermore, 41.5% (34/82) of HSCC specimens exhibited high LC3 immunoreactivity (adjacent non-cancerous tissues, 74.1%, 40/54; *P*=0.0002). Beclin-1 and LC3-II mRNA transcript levels were significantly lower in HSCCs than in paired non-cancerous tissues (*P*<0.0001, *P*=0.0001, respectively). Similarly, western blotting assays showed that beclin-1 and LC3-II were markedly decreased in HSCCs (*P*=0.02, *P*=0.004, respectively). A positive correlation was observed between the mRNA transcript levels of beclin-1 and LC3-II in HSCCs (r=0.51, *P*<0.0001; 95%CI: 0.273 to 0.689). Beclin-1 and LC3 expression were significantly correlated with T categories, differentiation and lymph node metastasis. Negative beclin-1 immuoreactivity and low LC3 expression were associated with poorer overall survival in HSCC patients (*P*<0.0001, *P*=0.0145, respectively). Multivariate analysis revealed that beclin-1 was an independent prognositic factor for overall survival.

**Conclusion:**

Beclin-1 and LC3-II are downregulated in HSCCs and their aberrant expression correlates with poor prognosis of HSCCs.

## Introduction

Autophagy, a type of non-apoptotic cell death, is characterized by the delivery of cytosolic materials and organelles to lysosomes for bulk degradation [1,2]. It is implicated in tumor growth and progression, and has been explored as a potential therapeutic target [3,4]. Approximately 30 genes have been identified to regulate autophagy in yeasts, with 16 homologues in humans [5]. Among these, beclin-1 and LC3 (microtubule-associated protein 1A/1B-light chain 3) play important roles in autophagy in mammalian cells [6]. Beclin-1 is a mammalian orthologue of the yeast Apg6/Vps30 gene [7], and beclin-1 functions as a scaffold for the formation of the PI3K complex, one of the first components recruited during the development of autophagosomes [8]. LC3 is a mammalian homologue of yeast Atg8 [9]. It is activated and processed by an ubiquitination-like reaction, and is regulated by Atg3 and Atg7 [9]. LC3 consists of a soluble form, LC3-I, with a molecular weight of 18 KD and a lipidated form, LC3-II, with a molecular weight of 16 KD, and has three isoforms, LC3A, LC3B, and LC3C in mammalian cells. LC3-II and the LC3B isoenzymes are implicated in autophagy [10,11]. Various stressors, such as hypoxia, starvation and chemotherapy, upregulate LC3 expression and promote the binding of cytosolic LC3-I to phosphatidylethanolamine to form autophagosome-specific LC3-II [10,12]. Therefore, LC3, especially the LC3-II is considered a reliable marker of autophagy.

Aberrant expression of beclin-1 and LC3 has been documented in several solid tumors, including human colon cancer [13], melanoma [14], ovarian cancer [15], lung cancer [16] and brain cancer [17]. Liang et al. reported reduced beclin-1 expression in human breast cancer tissues [18]. Clelia et al. revealed that beclin-1 was significantly decreased in human melanoma [14]. Furthermore, Beclin-1 down-regulation linked to autophagy defect may be associated with malignant phenotype and poor prognosis of hepatocellular carcinoma [19]. However, the expression of beclin-1 and LC3 has not been characterized in hypopharyngeal squamous cell carcinoma.

Hypopharyngeal cancers are usually squamous cell carcinomas, and have the worst prognosis among head and neck cancers. Despite the best therapeutic regimen currently available, The 5-year survival rate is estimated to be at 25% to 40% [20]. Lack of diagnostic and prognostic markers hampers the management of this dismal disease. We hypothesized that beclin-1 and LC3 are aberrantly expressed in hypopharyngeal squamous cell carcinomas. In the current study, we examined the expression of beclin-1 and LC3 at both the mRNA and protein levels in hypopharyngeal squamous cell carcinoma tissues and paired non-cancerous tissues and further studied whether beclin-1 and LC3 expression correlated with patient clinicopathological characteristics and prognostic factors.

## Materials and Methods

### Ethics Statement

The study protocol was approved by Ethics Boards of Qilu Hospital (the permit numbers is 12040), and tissue specimen acquisition was carried out in accordance with the institutional guidelines. All subjects signed written informed consent, and this consent procedure was approved by Ethics Boards of Qilu Hospital.

### Patients and tissue specimens

82 surgical hypopharyngeal squamous cell carcinoma specimens and 54 adjacent non-cancerous mucosal epithelial tissue specimens were obtained from patients who were admitted to the Department of Otolaryngology, Qilu Hospital, Jinan, China, between March 2009 and July 2011. The specimens were divided into two parts, one part was stored in liquid nitrogen immediately for total RNA and protein extraction, and the other was fixed in 10% buffered formaldehyde for immunohistochemistry. All subjects were smokers or drinkers. Patients who received neoadjuvant chemotherapy or radiotherapy were excluded from this study. Diagnosis and histological typing were carried out by two experienced pathologists according to the World Health Organization classification [21]. Tumor staging was based on the International Union against Cancer (UICC, 2002) TNM classification.

Patient demographic and clinicopathologic characteristics are shown in Table 1. The median age of the patients at the time of diagnosis was 59.5 (range, 38 to 76years) years with a male predominance (91.5%). Almost three quarters (75.6%) of the carcinomas were advanced stages (III-IV) and 72.0% had a moderate to poor degree of differentiation. All patients received post-operative radiotherapy in this study. The time of follow-up was calculated from the date of operation. The median patient follow-up duration was 24 months (range, 4-46 months).

**Table 1:** Patient demographic and clinicopathologic characteristics (n=82).

**Variable**	**No. (%)**
**Age at presentation**	
Median	59.5
Range	38-76
**Gender**	
Female	7 (8.5%)
Male	75 (91.5%)
**T category**	
T1/T2	27 (32.9%)
T3/T4	55 (67.1%)
**Node metastasis**	
N0	30 (36.6%)
N1+N2	52 (63.4%)
**M category**	
M0	82 (100%)
M1	0(0%)
**Clinical Stage**	
I+II	20 (24.4%)
III+IV	62 (75.6%)
**Histologic differentiation**	
Well	23 (28.0%)
Moderate	27 (33.0%)
Poor	32 (39.0%)

### Real-time reverse transcriptase polymerase chain reaction (RT-PCR)

Total RNA was extracted using Trizol reagent (Invitrogen, Carlsbad, CA). For first-strand cDNA synthesis, 1 µg RNA was reverse transcribed with 100 units of PrimerScript Reverse Transcriptase (Takara Bio, Dalian, China) and 50 µM Oligo dT Primer (Takara). Then, cDNAs were subjected to quantitative real-time PCR analysis. The sequences of the primers used in the current study were previously reported [19,22] and were as follows: beclin-1, 5’-AGCTGCCGTTATACTGTTCTG-3’ (sense), and 5’-ACTGCCTCCTGTGTCTTCAATCTT-3’ (antisense); LC3-II, 5’-GATGTCCGACTTATTCGAGAGC -3’ (sense) and

5’-TTGAGCTGTAAGCGCCTTCTA-3’ (antisense); GAPDH, 5’-TGAACGGGAAGCTCACTGG-3’ (sense) and 5’-TCCACCACCCTGTTGCTGTA-3’ (antisense) [19,22]. Real-time PCR was performed using an ABI 7900HT Sequence Detection system (ABI Applied Biosystems, Foster City, CA) in a 10-µl reaction containing 0.4 µM each primer, 1 µL template cDNA, 5 µL SYBR Premix EX *Taq* (Takara Bio), and 0.2 µL ROX reference dye. The PCR was run at 95°C for 30 sec followed by 40 cycles of 95°C for 5 sec and 60°C for 30 sec. All samples were analyzed in duplicate. GAPDH was used as an internal control. Gene expression was calculated using the comparative threshold cycle (2^-△△CT^) method.

### Western blotting assays

Proteins were extracted in RIPA lysis buffer containing 50 mM Tris (pH 7.4), 150 mM NaCl, 1% Triton X-100, 1% sodium deoxycholate, 0.1% SDS and protease inhibitor cocktail, and separated by 10% SDS-PAGE. The immunoblotting procedure was performed as previously described [23]. Briefly, Separated proteins were transferred to polyvinylidene fluoride membranes (Millipore, Boston, USA), and incubated with primary antibodies overnight at 4°C. The following antibodies (all from Abcam, Cambridge, UK) were used for the procedure: rabbit polyclonal anti-beclin-1 antibody (ab55878), rabbit polyclonal anti-LC3B antibody (ab63817) and mouse monoclonal anti-actin antibody (ab3280). Membranes were then incubated with horseradish peroxidase–conjugated antibodies (DAKO, Copenhagen, Denmark), and visualized using an Odyssey Infrared Imaging System (Li-Cor Biosciences, Lincoln, NE) and normalized against β-actin.

### Immunohistochemistry

For immunohistochemistry, 4-µm thick sections of 82 tumor tissues and 54 adjacent normal mucosal epithelial tissues were deparaffinized and rehydrated, and antigen retrieval was carried out by microwave treatment of the slides in 50 mM Tris-EDTA (pH 9.0) for 20 min. Endogenous peroxidase was quenched with 3% hydrogen peroxide for 15 min at room temperature. Non-specific binding was blocked with 10% normal goat serum for 15 min. The slides were incubated with rabbit polyclonal anti-beclin-1 antibody (ab55878) or anti-LC3B antibody (ab63817) (both from Abcam) overnight at 4°C, respectively. Sections were then incubated with a biotinylated secondary antibody (Jingmei Biotech, Jinan, China) in 37°C for 15 min, and then with peroxidase-conjugated streptavidin for 15 min. Chromogenic reaction was carried out in 3,3'-diaminobenzidine tetrahydrochloride (Zsbio, Beijing, China) for 5 min. Slides were counterstained with hematoxylin. For negative controls, the primary antibody was omitted.

Staining was evaluated under a light microscope independently by two experienced pathologists blinded to patient clinicopathologic data. Beclin-1 reactivity was expressed as granular and diffuse cytoplasmic and membranous staining and evaluated according to intensity and proportion. The intensity of staining was scored as follows: 0, negative; 1, weak; 2, moderate; 3, strong. The percentages of beclin-1 positive cells were recorded as previously reported [24] into five categories: 0 (≤5%), 1 (6–25%), 2 (26–50%), 3 (51–70%) and 4 (>70%). The index of immunoreactivity was calculated by adding the intensity score and the proportion scores. Beclin-1 positivity was defined as an immunoreactive score of at least 4. LC3 positivity was defined as brown staining in the cytoplasm and occasionally in the nuclei. The proportion of LC3 positive cells were scored into four categories: 0 (≤5%), 1 (6%-25%), 2 (26%-50%), and 3 (>50%). A score of 0 to 1 was defined as low expression, and a score of 2 to 3 as high expression. The photographs were taken using a Leica digital microscope color camera (DFC295, Germany) at 20 x-magnification of objective lens by Leica light microscope (DM2500, Germany) with a total magnification of 200 x.

### Statistical analysis

Statistical analysis was performed using the GraphPad Prism 5.0 (GraphPad Software Inc., USA) and SPSS 16.0 (SPSS Inc., USA). Wilcoxon matched pairs test was used to compare mRNA and protein of LC3 and beclin-1 in tumor vs. non-tumor tissues. The relationship between categorical variables was analyzed by Chi-square test or Fisher’s exact test. Spearman’s test for non-normal correlation coefficients was used to evaluate the correlation between beclin-1 and LC3 expression. Overall survival curves were drawn using Kaplan-Meier method, and differences between the curves were analyzed by the log-rank test. Univariate and multivariate analyses of prognostic factors were performed by the Cox proportional hazards regression model. For all tests, a two-sided *P* value <0.05 was considered statistically significant.

## Results

### Beclin-1 and LC3-II are downregulated in hypopharyngeal squamous cell carcinoma tissues

**Figure 1:**
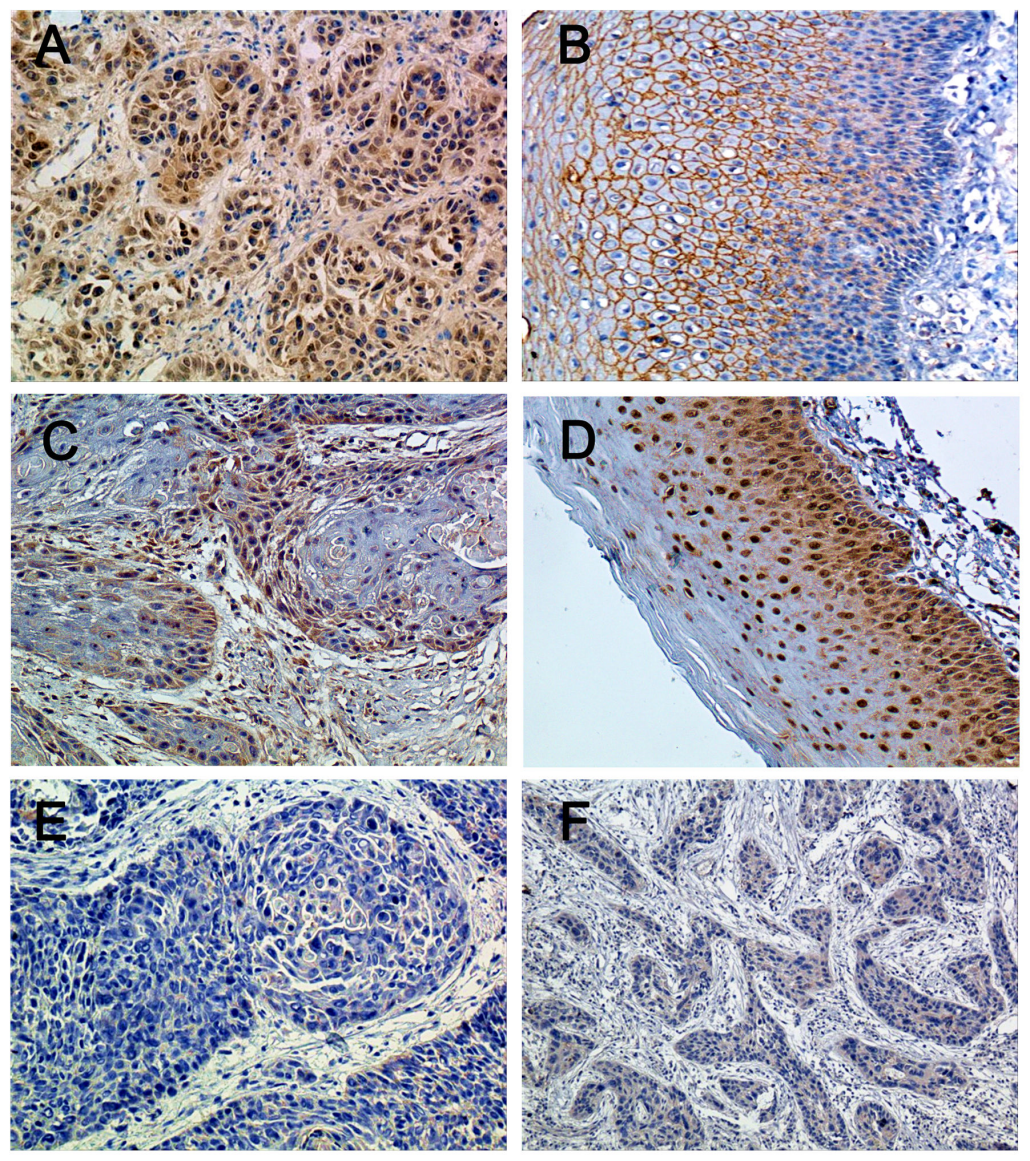
**Representative images of beclin-1 and LC3 immunohistochemical staining.**

We examined the expression of beclin-1 and LC3 in hypopharyngeal squamous cell carcinoma tissues by immunohistochemistry. Beclin-1 and LC3 were observed mainly in the cytoplasms or cytomembranes of hypopharyngeal squamous cell carcinoma cells and adjacent normal squamous epithelial cells. In addition, LC3 was occasionally found in the nuclei of cancer cells and normal epithelial cells. Beclin-1 was positively expressed in 42.7% (35/82) of the hypopharyngeal squamous cell carcinoma specimens, which was markedly lower than that of adjacent non-cancerous tissues (79.6%, 43/54) (*P*<0.0001) (Figure 1A and 1B and Table 2). Furthermore, high LC3 immunoreactivity was 41.5% (34/82) in the hypopharyngeal squamous cell carcinoma specimens as compared with 74.1% (40/54) in adjacent non-cancerous tissues (*P*=0.0002) (Figure 1C and 1D). These findings suggest that beclin-1 and LC3 expression is significantly downregulated in hypopharyngeal squamous cell carcinoma tissues.

Tissue specimens were immunohistochemically stained for beclin-1 and LC3 as detailed in Methods. Beclin-1 reactivity was expressed as granular and diffuse cytoplasmic and membranous staining and LC3 positivity was defined as brown staining in the cytoplasm and occasionally in the nuclei. (A) Positive expression of beclin-1 in hypopharyngeal squamous cell carcinoma cells. (B) Positive expression of beclin-1 in adjacent normal mucosal epithelial cells. (C) High expression of LC3 in hypopharyngeal squamous cell carcinoma cells. (D) High expression of LC3 in adjacent normal mucosal epithelial cells. (E) Negative expression of beclin-1 in hypopharyngeal squamous cell carcinoma cells. (F) Low expression of LC3 in hypopharyngeal squamous cell carcinoma cells. Original magnification: 200 x.

**Table 2:** Expression patterns of beclin-1 and LC3 in hypopharyngeal squamous cell carcinoma (HSCC) and adjacent non-tumor tissues.

Group	No.	Beclin-1 expression		*P* value	LC3 expression		*P* value
		Positive	Negative		High	Low	
HSCC tissues	82	35	47	<0.0001	34	48	0.0002
Normal tissues	54	43	11		40	14	

*P* values were determined by Chi-square tests

**Figure 2:**
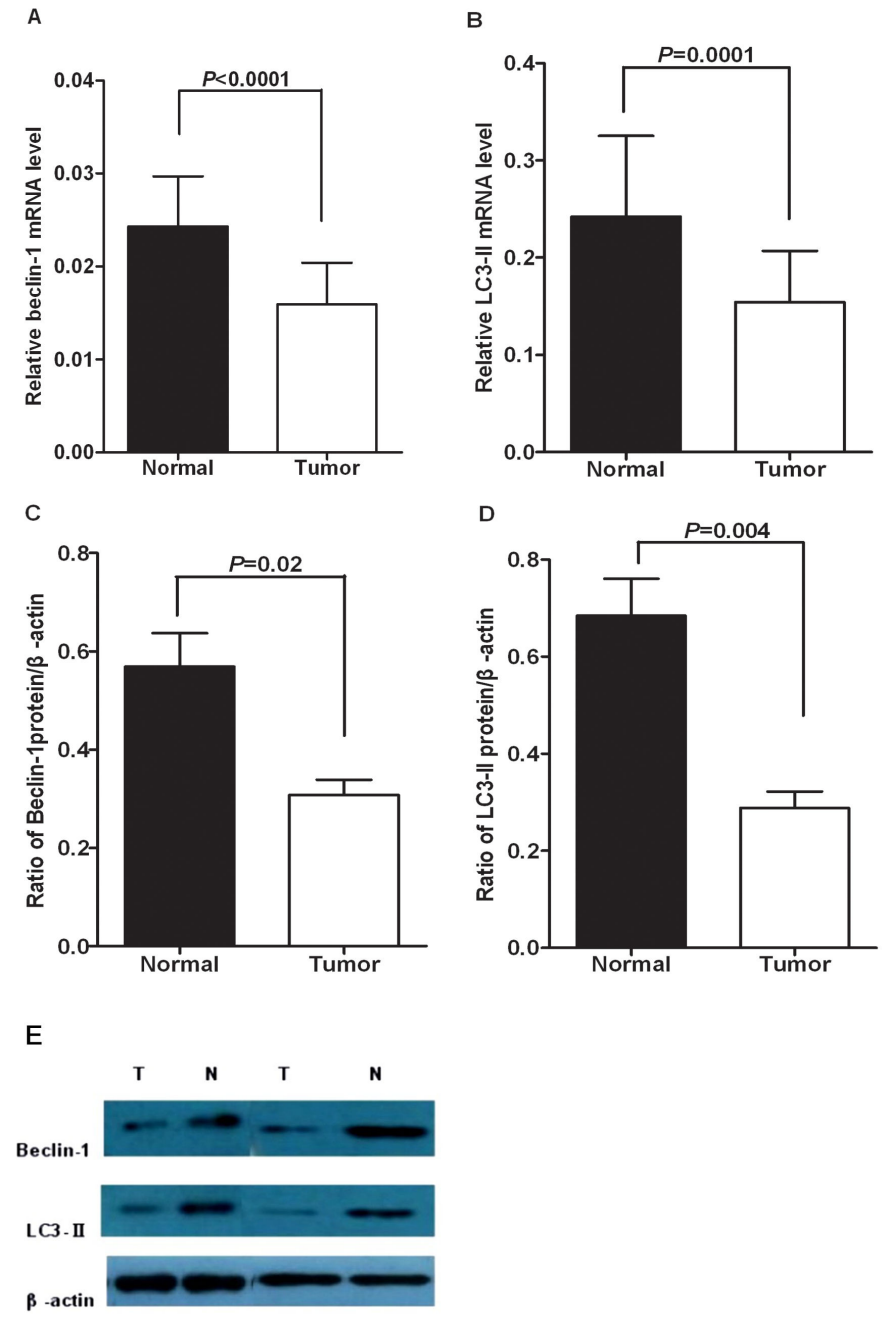
**Expression of beclin-1 and LC3-II in human hypopharyngeal squamous cell carcinoma (HSCC) tissues.**

To confirm the above findings, we further determined the expression of beclin-1 and LC3 in 54 paired hypopharyngeal squamous cell carcinoma tissues and adjacent normal tissues. Our RT-PCR assays showed that the mean mRNA transcript levels of beclin-1 were 0.016±0.004 in hypopharyngeal squamous cell carcinoma tissues and 0.024±0.005 in the adjacent normal tissues (*P*<0.0001) (Figure 2A). The mean mRNA transcript levels of LC3-II were 0.154±0.053 in hypopharyngeal squamous cell carcinoma tissues and 0.242±0.083 in the adjacent normal tissues (*P*=0.0001) (Figure 2B). A positive correlation was observed between the mRNA transcript levels of beclin-1 and LC3 in hypopharyngeal squamous cell carcinoma tissues (r=0.51, *P*<0.0001; 95%CI: 0.273 to 0.689).

Data were shown as mean±SEM. The mRNA levels of (A) beclin-1and (B) LC3-II were detected by real-time PCR in 54 HSCC tissues and matched adjacent non-tumor tissues. The protein levels of (C) beclin-1 and (D) LC3-II were analyzed by Western blotting assays of 10 paired HSCC tissues. β-actin was used as an internal control. (E) Relative low levels of beclin-1 and LC3-II protein were found in tumor tissues.

We further examined the expression of beclin-1 and LC3-II by immunoblotting assays. Similarly, the protein level of beclin-1 was markedly lower in the hypopharyngeal squamous cell carcinoma tissues (0.308±0.031) than in paired normal tissue (0.569±0.068) (*P*=0.02) (Figure 2C). Additionally, the protein level of LC3-II was also markedly lower in the hypopharyngeal squamous cell carcinoma tissues (0.289±0.034) than in paired normal tissue (0.685±0.076) (*P*=0.004) (Figure 2D).

### Low beclin-1 and LC3 expression correlates with tumor stage and lymph node metastasis

We analyzed whether beclin-1 and LC3 expression in hypopharyngeal squamous cell carcinoma tissues correlated with patient clinicopathologic characteristics. The associations between beclin-1 and LC3 expression and clinicopathologic factors are presented in Table 3. Significant correlation was found between negative beclin-1 expression and advanced clinical stage (r=0.299, *P*=0.005), increasing T stage (r=0.365, *P*=0.0004), poor differentiation (r=0.392, *P*=0.0006) and cervical lymph node metastases (r=0.302, *P*=0.004). Similarly, LC3 level decreased in hypopharyngeal squamous cell carcinoma tissues of patients with advanced T stage (r=0.245, *P*=0.022), poor differentiation (r=0.504, *P*<0.0001) and cervical lymph node metastases (r=0.275, *P*=0.01).

**Table 3:** Beclin-1 and LC3 expression and patient clinicopathologic characteristics.

Characteristics	No.	Beclin-1 expression		*P* value	LC3 expression		*P* value
		Positive	Negative		High	Low	
**Gender**				1.000			0.694
Male	75	32	43		32	43	
Female	7	3	4		2	5	
**Age, years**				0.264			0.073
<60	41	15	26		13	28	
≥60	41	20	21		21	20	
**Clinical stage**				0.005^*^			0.053
I–II	20	14	6		12	8	
III-IV	62	21	41		22	40	
**T stage**				0.0004^*^			0.022^*^
T1-T2	27	19	8		16	11	
T3-T4	55	16	39		18	37	
**Differentiation**				0.0006^*^			<0.0001^*^
Well	23	17	6		20	3	
Moderate	27	11	16		8	19	
Poor	32	7	25		6	26	
**Node metastasis**				0.004^*^			0.010^*^
N0	30	19	11		18	12	
N1-N2	52	16	36		16	36	

*P* values were determined by Chi-square tests or Fisher’s exact tests

^*^ *P*<0.05

### Association of beclin-1 expression, LC3 level, and overall survival

**Figure 3:**
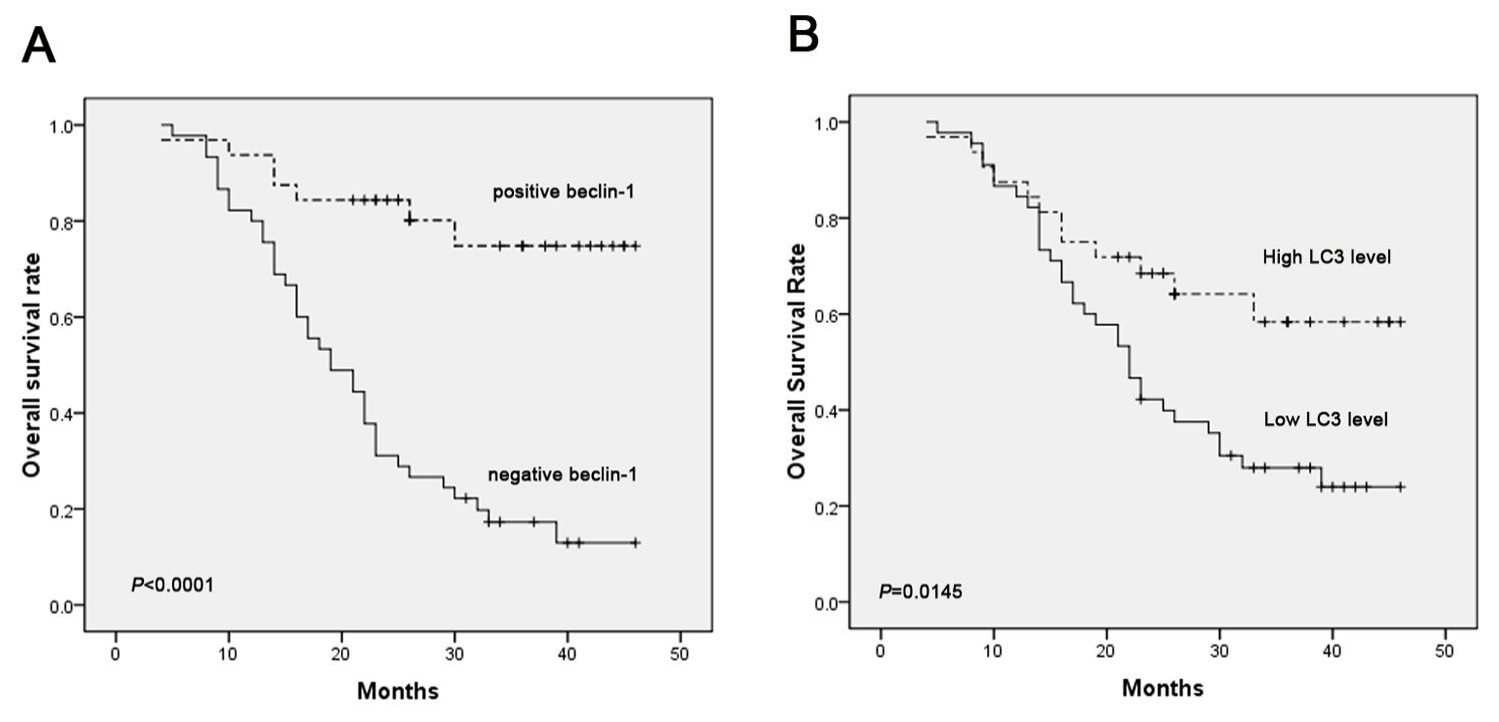
**Kaplan-Meier curves for beclin-1 and LC3 expression in 82 patients with hypopharyngeal squamous cell carcinoma (HSCC)**. (A) Negative expression of beclin-1 was associated with poor prognosis (*P*<0.0001). (B) Low level of LC3 was associated with poor prognosis (*P*=0.0145).

To investigate whether beclin-1 and LC3 could be predictive for the overall survival of hypopharyngeal squamous cell carcinoma patients, the Kaplan-Meier analysis was performed, which showed that the overall survival rates of patients with beclin-1 negative tumors was significantly lower than of patients with beclin-1 positive tumors (*P*<0.0001) (Figure 3A). The 2-year overall survival rates for beclin-1 negative and beclin-1 positive patients were 31.1% and 84.4%, respectively. Furthermore, patients with low LC3 level had a significantly poorer prognosis than high LC3 patients (*P*=0.0145) (Figure 3B). The 2-year overall survival rates with low and high level of LC3 were 42.2% and 68.5%, respectively. Univariate Cox regression analysis summarized in Table 4 showed that clinical stage (*P*=0.0009), T stage (*P*=0.0038), lymph node metastasis (*P*=0.0007), beclin-1 expression (*P*<0.0001) and LC3 level (*P*=0.018) were significantly associated with overall survival. In the multivariate Cox regression analysis, beclin-1 expression was an independent prognostic factor (hazard ratio 6.107, *P*=0.0004, 95%CI: 2.238 to 16.666) (Table 4). However, LC3 level and clinical characteristics including clinical stage, T stage, differentiation and lymph node metastasis were not determined as independent prognostic indicators.

**Table 4:** Results of univariate and multivariate Cox regression analyses in 82 patients^a^.

Parameter	Unfavorable vs. favorable	Univariate analysis			Multivariate analysis		
		HR	95% CI	P value	HR	95% CI	P value
Clinical stage	III-IV vs. I–II	7.412	2.274-24.163	0.0009*	4.439	0.896-21.997	0.068
T stage	T3-T4 vs T1-T2	3.034	1.43-6.438	0.0038*	0.826	0.325-2.098	0.6871
Differentiation	poor vs. well and moderate	1.369	0.755-2.482	0.3009	1.163	0.71-1.907	0.5481
Node metastasis	N1-N2 vs. N0	3.823	1.765-8.281	0.0007*	1.34	0.496-3.619	0.5632
beclin-1 expression	Negative vs. Positive	6.353	2.787-14.48	<.0001*	6.107	2.238-16.666	0.0004*
LC3 level	Low vs. High	2.294	1.153-4.563	0.018*	0.623	0.261-1.488	0.2871

^a^ adjusted for age and gender groups. HR, hazard ratio; CI, confidence interval.

* *P*<0.05

## Discussion

In the present study, we revealed for the first time that both beclin-1 and LC3-II are significantly downregulated in hypopharyngeal squamous cell carcinoma tissues compared to adjacent normal mucosal epithelium tissues. Beclin-1 and LC3 were mainly located in the cytoplasm or on the cell membrane in both hypopharyngeal squamous cell carcinoma cells and squamous epithelial cells in adjacent non-cancerous tissue with occasional expression of LC3 in the nuclei. Autophagy is implicated in tumorigenesis and tumor development [25]. Beclin-1 plays an important role in autophagy, differentiation and apoptosis of cancer cells [26]. Liang et al. reported lower expression of beclin-1 in human breast cancer cell lines and tissue [18]. Clelia et al. reported decreased beclin-1 mRNA and protein in human melanoma [14]. The loss of beclin-1 expression was correlated with poor prognosis in colonic carcinomas [13], lymphomas [24] and hepatocellular cancers [19]. In contrast, overexpression of beclin-1 has been reported to correlate with progression of gastric and colorectal cancer [27]. Giatromanolaki et al. reported high beclin-1 expression was predictor of poor prognosis in endonetrial adenocarcinomas [8].

In this study, we found lower expression of beclin-1 in hypopharyngeal squamous cell carcinoma tissues vs. adjacent non-cancerous tissues at both the mRNA and protein levels. We further demonstrated that decreased expression of beclin-1 has been correlated with advanced clinical stage and T stage, poor differentiation, and lymph node metastasis. Moreover, survival analysis showed that tumors with negative beclin-1 expression had a significantly poorer overall survival, beclin-1 was an independent prognositic factor. Therefore, these results might help us to determin the prognosis of patients according to the expression of beclin-1. Several mechanisms that describe how autophagy induced by beclin-1 prevents cancer can be considered. First, there is death by autophagy. Second, autophagy can eliminate damaged organelles to remove the sources of genotoxic materials including damaged DNA products, which prevent oxidative stress from damaging the organelles. The inhibition of tumorigenesis by autophagy is also derived from the capacity to negatively regulate the proliferation of cells [18].

The lipidated form of LC3 (LC3-II) is recruited to the autophagosomes [10]. Consistently, we found that low LC3-II expression has significant association with more advanced T stage, poor differentiation, lymph node metastasis and poor prognosis in HSCC patients. Additionally, the mRNA and protein levels of LC3-II were lower in hypopharyngeal squamous cell carcinoma tissues than in adjacent non-cancerous tissues. This finding is consistent with previous findings in brain and ovary cancers [15,28]. However, Yoshioka et al. reported increased expression of LC3 in esophageal and gastrointestinal neoplasms [29]. We therefore speculate that the status and role of LC3 expression vary across tumor types. Further research should be directed at determining whether and how the autophagy gene LC3 inhibits or promotes tumorigenesis.

Thus far, much of research on hypopharyngeal cancers is focus on apoptosis. Apoptosis and autophagy are both self-destructive processes. Both have been considered as programmed cell death, with apoptotic cell death labeled as type I and autophagic cell death as type II cell death [29]. Recent evidence suggests that autophagy and apoptosis are regulated by common upstream signals and share common components (e.g., the Bcl-2 family members and P13K/Akt signaling pathway) [30,31]. With regards to cancer development and treatment, inhibiting apoptosis may cause autophagy in some cancer cells [32], but triggers apoptosis in other cancer cells [33]. Recent studies have shown that defective autophagy synergized with defective apoptosis results in increased DNA damage and genomic instability that ultimately facilitates tumor progression. Therefore, autophagy and apoptosis may play an important role in tumorigenesis and tumor progression [19]. In the present study, for the first time, we revealed the potential prognostic significance of autophagy genes in hypopharyngeal squamous cell carcinoma.

## Conclusions

We demonstrated that the expression of both beclin-1 and LC3-II are significantly lower in hypopharyngeal squamous cell carcinoma tissues than in adjacent non-cancerous tissues. Furthermore, beclin-1 and LC3 expression was associated with certain clinical characteristics such as tumor stage, differentiation and lymph node metastasis, and had significant impacts on the prognosis of hypopharyngeal squamous cell carcinoma patients, beclin-1 was an independent prognositic factor for overall survival. These results suggest that the autophagic genes beclin-1 and LC3 play an important role in the progression and prognosis of hypopharyngeal squamous cell carcinoma, and could be novel therapeutic targets for future treatment of human hypopharyngeal squamous cell carcinomas. However, further studies of larger scale are necessary to verify the preliminary findings in this study.

## References

[1] Klionsky DJ (2007) Autophagy: from phenomenology to molecular understanding in less than a decade. Nat Rev Mol Cell Biol 8: 931-937. doi:10.1038/nrm2245. PubMed: 17712358. 17712358 10.1038/nrm2245

[2] Kroemer G, Jäättelä M (2005) Lysosomes and autophagy in cell death control. Nat Rev Cancer 5: 886-897. doi:10.1038/nrc1738. PubMed: 16239905. 16239905 10.1038/nrc1738

[3] Eisenberg-Lerner A, Kimchi A (2009) The paradox of autophagy and its implication in cancer etiology and therapy. Apoptosis 14: 376-391. doi:10.1007/s10495-008-0307-5. PubMed: 19172397. 19172397 10.1007/s10495-008-0307-5

[4] Zois CE, Koukourakis MI (2009) Radiation-induced autophagy in normal and cancer cells: towards novel cytoprotection and radio-sensitization policies? Autophagy 5: 442-450. doi:10.4161/auto.5.4.7667. PubMed: 19164950. 19164950 10.4161/auto.5.4.7667

[5] Cheong H, Klionsky DJ (2008) Biochemical methods to monitor autophagy-related processes in yeast. Methods Enzymol 451: 1-26. doi:10.1016/S0076-6879(08)03201-1. PubMed: 19185709. 19185709 10.1016/S0076-6879(08)03201-1

[6] Eskelinen EL, Saftig P (2009) Autophagy: a lysosomal degradation pathway with a central role in health and disease. Biochim Biophys Acta 1793: 664-673. doi:10.1016/j.bbamcr.2008.07.014. PubMed: 18706940. 18706940 10.1016/j.bbamcr.2008.07.014

[7] Cao Y, Klionsky DJ (2007) Physiological functions of Atg6/Beclin 1: a unique autophagy-related protein. Cell Res 17: 839-849. doi:10.1038/cr.2007.78. PubMed: 17893711. 17893711 10.1038/cr.2007.78

[8] Giatromanolaki A, Koukourakis MI, Koutsopoulos A, Chloropoulou P, Liberis V et al. (2011) High Beclin 1 expression defines a poor prognosis in endometrial adenocarcinomas. Gynecol Oncol 123: 147-151. doi:10.1016/j.ygyno.2011.06.023. PubMed: 21741077. 21741077 10.1016/j.ygyno.2011.06.023

[9] Tanida I, Tanida-Miyake E, Ueno T, Kominami E (2001) The human homolog of Saccharomyces cerevisiae Apg7p is a Protein-activating enzyme for multiple substrates including human Apg12p, GATE-16, GABARAP, and MAP-LC3. J Biol Chem 276: 1701-1706. PubMed: 11096062. 11096062 10.1074/jbc.C000752200

[10] Kabeya Y, Mizushima N, Ueno T, Yamamoto A, Kirisako T et al. (2000) LC3, a mammalian homologue of yeast Apg8p, is localized in autophagosome membranes after processing. EMBO J 19: 5720-5728. doi:10.1093/emboj/19.21.5720. PubMed: 11060023. 11060023 10.1093/emboj/19.21.5720 PMC305793

[11] Klionsky DJ, Abeliovich H, Agostinis P, Agrawal DK (2008) Guidelines for the use and interpretation of assays for monitoring autophagy in higher eukaryotes. Autophagy 4: 151-175. PubMed: 18188003. 18188003 10.4161/auto.5338 PMC2654259

[12] Mizushima N, Yamamoto A, Matsui M, Yoshimori T, Ohsumi Y (2004) In vivo analysis of autophagy in response to nutrient starvation using transgenic mice expressing a fluorescent autophagosome marker. Mol Biol Cell 15: 1101-1111. PubMed: 14699058. 14699058 10.1091/mbc.E03-09-0704 PMC363084

[13] Li BX, Li CY, Peng RQ, Wu XJ, Wang HY et al. (2009) The expression of beclin 1 is associated with favorable prognosis in stage IIIB colon cancers. Autophagy 5: 303-306. doi:10.4161/auto.5.3.7491. PubMed: 19066461. 19066461 10.4161/auto.5.3.7491

[14] Miracco C, Cevenini G, Franchi A, Luzi P, Cosci E et al. (2010) Beclin 1 and LC3 autophagic gene expression in cutaneous melanocytic lesions. Hum Pathol 41: 503-512. doi:10.1016/j.humpath.2009.09.004. PubMed: 20004946. 20004946 10.1016/j.humpath.2009.09.004

[15] Shen Y, Li DD, Wang LL, Deng R, Zhu XF (2008) Decreased expression of autophagy-related proteins in malignant epithelial ovarian cancer. Autophagy 4: 1067-1068. PubMed: 18776739. 18776739 10.4161/auto.6827

[16] Jiang ZF, Shao LJ, Wang WM, Yan XB, Liu RY (2012) Decreased expression of Beclin-1 and LC3 in human lung cancer. Mol Biol Rep 39: 259-267. doi:10.1007/s11033-011-0734-1. PubMed: 21556768. 21556768 10.1007/s11033-011-0734-1

[17] Miracco C, Cosci E, Oliveri G, Luzi P, Pacenti L et al. (2007) Protein and mRNA expression of autophagy gene Beclin 1 in human brain tumours. Int J Oncol 30: 429-436. PubMed: 17203225. 17203225

[18] Liang XH, Jackson S, Seaman M, Brown K, Kempkes B et al. (1999) Induction of autophagy and inhibition of tumorigenesis by beclin 1. Nature 402: 672-676. doi:10.1038/45257. PubMed: 10604474. 10604474 10.1038/45257

[19] Ding ZB, Shi YH, Zhou J, Qiu SJ, Xu Y et al. (2008) Association of autophagy defect with a malignant phenotype and poor prognosis of hepatocellular carcinoma. Cancer Res 68: 9167-9175. doi:10.1158/0008-5472.CAN-08-1573. PubMed: 19010888. 19010888 10.1158/0008-5472.CAN-08-1573

[20] Wycliffe ND, Grover RS, Kim PD, Simental A Jr (2007) Hypopharyngeal Cancer. Top Magn Reson Imaging 18: 243-258. doi:10.1097/RMR.0b013e3181570c3f. PubMed: 17893590. 17893590 10.1097/RMR.0b013e3181570c3f

[21] Shanmugaratnam K, Sobin LH (1993) The World Health Organization histological classification of tumours of the upper respiratory tract and ear Cancer 71: 387-392. 10.1002/1097-0142(19930415)71:8<2689::aid-cncr2820710843>3.0.co;2-h 8453591

[22] Tu YF, Kaipparettu BA, Ma Y, Wong LJ (2011) Mitochondria of highly metastatic breast cancer cell line MDA-MB-231 exhibits increased autophagic properties. Biochim Biophys Acta 1807: 1125-1132. doi:10.1016/j.bbabio.2011.04.015. PubMed: 21570379. 21570379 10.1016/j.bbabio.2011.04.015

[23] Liang C, Guo S, Yang L (2013) All-trans retinoic acid upregulates VEGF expression in glioma cells in vitro. J Biomed Res 27: 51-55. doi:10.7555/JBR.27.20120048. PubMed: 23554794. 23554794 10.7555/JBR.27.20120048 PMC3596755

[24] Huang JJ, Li HR, Huang Y, Jiang WQ, Xu RH et al. (2010) Beclin 1 expression: a predictor of prognosis in patients with extranodal natural killer T-cell lymphoma, nasal type. Autophagy 6: 777-783. doi:10.4161/auto.6.6.12784. PubMed: 20639699. 20639699 10.4161/auto.6.6.12784

[25] Lomonaco SL, Finniss S, Xiang C, Decarvalho A, Umansky F et al. (2009) The induction of autophagy by gamma-radiation contributes to the radioresistance of glioma stem cells. Int J Cancer 125: 717-722. doi:10.1002/ijc.24402. PubMed: 19431142. 19431142 10.1002/ijc.24402

[26] Liang XH, Kleeman LK, Jiang HH, Gordon G, Goldman JE et al. (1998) Protection against fatal Sindbis virus encephalitis by beclin, a novel Bcl-2-interacting protein. J Virol 72: 8586-8596. PubMed: 9765397. 9765397 10.1128/jvi.72.11.8586-8596.1998 PMC110269

[27] Ahn CH, Jeong EG, Lee JW, Kim MS, Kim SH et al. (2007) Expression of beclin-1, an autophagy-related protein, in gastric and colorectal cancers. APMIS 115: 1344-1349. doi:10.1111/j.1600-0463.2007.00858.x. PubMed: 18184403. 18184403 10.1111/j.1600-0463.2007.00858.x

[28] Aoki H, Kondo Y, Aldape K, Yamamoto A, Iwado E et al. (2008) Monitoring autophagy in glioblastoma with antibody against isoform B of human microtubule-associated protein 1 light chain 3. Autophagy 4: 467-475. PubMed: 18259115. 18259115 10.4161/auto.5668

[29] Yoshioka A, Miyata H, Doki Y, Yamasaki M, Sohma I et al. (2008) LC3, an autophagosome marker, is highly expressed in gastrointestinal cancers. Int J Oncol 33: 461-468. PubMed: 18695874. 18695874

[30] Coates JM, Galante JM, Bold RJ (2010) Cancer therapy beyond apoptosis: autophagy and anoikis as mechanisms of cell death. J Surg Res 164: 301-308. doi:10.1016/j.jss.2009.07.011. PubMed: 20031162. 20031162 10.1016/j.jss.2009.07.011

[31] Djavaheri-Mergny M, Maiuri MC, Kroemer G (2010) Cross talk between apoptosis and autophagy by caspase-mediated cleavage of Beclin 1. Oncogene 29: 1717-1719. doi:10.1038/onc.2009.519. PubMed: 20101204. 20101204 10.1038/onc.2009.519

[32] Degenhardt K, Mathew R, Beaudoin B, Bray K, Anderson D et al. (2006) Autophagy promotes tumor cell survival and restricts necrosis, inflammation, and tumorigenesis. Cancer Cell 10: 51-64. doi:10.1016/j.ccr.2006.06.001. PubMed: 16843265. 16843265 10.1016/j.ccr.2006.06.001 PMC2857533

[33] Amaravadi RK, Yu D, Lum JJ, Bui T, Christophorou MA et al. (2007) Autophagy inhibition enhances therapy-induced apoptosis in a Myc-induced model of lymphoma. J Clin Invest 117: 326-336. doi:10.1172/JCI28833. PubMed: 17235397. 17235397 10.1172/JCI28833 PMC1765515

